# The Influence of Temperature Differences in Smoking Chamber and Furnace and Smoking Time on the Quality of Medium-Ground Sausages

**DOI:** 10.3390/molecules25235515

**Published:** 2020-11-25

**Authors:** Paulina Duma-Kocan, Mariusz Rudy, Marian Gil, Renata Stanisławczyk

**Affiliations:** Department of Agricultural Processing and Commodity Science, Institute of Food and Nutrition Technology, College of Natural Sciences, University of Rzeszow, Zelwerowicza 4, 35-601 Rzeszów, Poland; mrudy@ur.edu.pl (M.R.); mgil@ur.edu.pl (M.G.); rstanisl@univ.rzeszow.pl (R.S.)

**Keywords:** medium-ground sausages, traditional smoking, texture, colour

## Abstract

The aim of the work was to determine the impact of two variants (A and B) of smoking (differing in temperature values, furnace, top and bottom of the smoking chamber and the length of smoking time) in the sausage technological process carried out in a traditional smoking chamber with an indirect furnace. The research material consisted of medium-ground sausages: Country, Home and Bieszczady sausages. The research showed that, as the temperature in the smoking chamber and the smoking time changed, the following texture parameters decreased: cycle hardness 1 and 2, springiness, gumminess and chewiness. In addition, there were shown statistically significant differences (*p* < 0.05) in the chemical composition of Country sausage between the applied smoking variants. It was also found that the temperature of the furnace, upper and lower smoking chamber and the length of smoking time did not have a statistically significant influence on the share of red (a*) and yellow (b*) in the analysed sausages.

## 1. Introduction

Meat and meat products are the main source of full-value protein. In addition, they contain vitamins and minerals that are an important element of the human diet [1,2,3,4].

One of the basic technological processes in the production of sausages is heat treatment. The most common types of such treatments are smoking, steaming or baking. This can mainly refer to products obtained by traditional methods. Thermal meat processing is essential to get a tasty and safe product. Heat treatment strongly affects the texture; protein and other important quality factors, such as the colour, flavour and juiciness of the final product [5,6,7,8]. Since texture is considered to be one of the most important indicators of food product quality, it is important to understand the physical changes of meat texture during heating. Protein denaturation reduces the water-holding capacity, shrinks muscle fibres and causes connective tissue degradation, subsequently leading to a harder texture [9]. The denaturation of actin and myofibrillar proteins decreases tenderness, while the gelatinisation of connective tissue makes the meat tender again [10]. Dominguez et al.’s research [11,12] showed that there is a direct relationship between the type of heat treatment and the formation of volatile compounds that can affect consumer acceptance. The type of heat treatment, as well as the way it is carried out, can affect the chemical composition of meat due to nutrient losses [13,14,15].

Smoking is one of the oldest methods of meat product technology [16,17]. It is defined as subjecting meat products to smoke, which is formed in the process of the thermal decomposition (pyrolysis) of wood of various types of deciduous trees. Smoke is generated as a result of incomplete wood burning [18]. The chemical compounds contained in smoke perform preserving (antioxidative and bacteriostatic), flavouring, dyeing and impregnating functions [19,20]. Smoking has a significant impact on the quality of the final product. It increases the sensory qualities of meat and extends the shelf life of smoked meats without spoiling their quality [21]. Meat products preserved in this way have special physicochemical properties—due to which, they become less susceptible to bacterial decomposition. Properly conducted smoking is also conductive to the elimination of pathogenic microflora, which is a potential threat to consumer health [21,22,23].

The properties of the smoked product depend on many factors involved in the smoking process—namely, the temperature of the product, the way it is prepared, the state before smoking, etc. Current knowledge about smoke production and the smoking process allows to control the quality of the smoked product. The most important criteria for assessing smoked products by the consumer are the uniformity of smoking, colour, taste, smell and durability of the product [24].

In the specialist literature, there is no research on the influence of various smoking variants (differing in temperature values of the furnace, at the top and bottom of the smoking chamber and the length of smoking time) in the technological process of traditional medium-ground sausages carried out in a traditional smoking chamber with an indirect furnace on the chemical composition, texture and colour parameters of these products.

In this connection, the purpose of the work was to determine the influence of two variants (A and B) of smoking (differing in temperature value, grate, top and bottom of the smoking chamber and the length of smoking time) in the technological process of traditional medium-ground sausages carried out in a traditional smoking chamber with an indirect furnace on the chemical composition, texture and colour parameters of these products.

## 2. Results and Discussion

The texture is probably the most important quality factor associated with consumer satisfaction in consuming meat products [25]. According to De Man [26], the texture can be defined as “the way in which the structural components of food could be arranged into micro and macrostructure and the external manifestations for that structure”.

Table 1 presents the results regarding the texture parameters of medium-ground sausages.

Among the texture parameters that were tested, hardness is the most important parameter to consumers, as it determines the commercial value of meat products [27]. Hardness can be related to the force necessary to break the food with the incisors during mastication. The conducted study showed a statistically significant (*p* < 0.05) impact of the smoking variant on the differentiation of texture parameters, such as cycle hardness 1 and 2, rigidity, gumminess and chewiness, but only in the case of Bieszczady sausage. Higher values of these features were found when the smoking variant A of Bieszczady sausage was used. In addition, statistically significant differences (*p* < 0.05) were found for the cohesiveness and resilience of Home sausage, where higher values of these texture parameters were demonstrated when using smoking variant B. In the case of using smoking variant B in Home and Country sausages in the technological processes, slightly lower values of hardness, gumminess and chewiness were obtained; however, the differences for these features were not statistically significant. Another indicator that forms a texture profile is springiness. It can be defined as the elasticity, expressed in (mm)—it is the possibility of returning of the tested sample from a deformed state to the initial state [28]. For this parameter, no statistically significant differences were found in the tested sausages depending on the smoking variant used. Adhesiveness can be defined as the force needed to overcome the forces occurring between food and the material in close contact with it [29]. This force depends on the viscoelastic properties and viscosity of the substances tested, as well as the interaction of the adhesiveness and cohesiveness forces [30]. The temperature and duration of smoking did not have a significant impact on the value of the adhesiveness. The average values of this parameter among the tested sausages were 0.18–1.65 mJ. Chewiness is the work necessary to destroy the internal bonds of the test sample—in this case, medium-ground sausage samples. It is a secondary parameter that depends on the hardness, cohesiveness and springiness [31]. In this study, it was observed that, together with the temperature change in the smoking chamber and the smoking time, the parameters of springiness, gumminess and chewiness decreased.

Bhuyan et al. [32], analysing the impacts of various smoking methods on the quality of pork sausages, did not find statistically significant differences (*p* ≥ 0.05) in terms of TPA parameters. The quoted authors showed higher values regarding the hardness, chewiness, cohesiveness and resilience in the case of hot smoked sausages. Similar reports were also made by Martinez et al. [33], who studied the effects of two commercial liquid smoke (LS) flavourings on the texture of salted pork loin and salted bacon and reported the lowest values for hardness, fracturability, cohesiveness, gumminess and chewiness for bacon treated with liquid smoke (LS). Additionally, Yang et al. [34] in their studies did not show statistically significant differences (*p* > 0.05) between roasted and smoked sausages in relation to such texture parameters as cohesiveness, resilience, chewiness and springiness. In studies conducted by Abdulhameed et al. [35] on chicken sausage, it was shown that with the increasing temperature and time during steaming, the values of the following texture parameters, i.e., hardness, gumminess, chewiness and cohesiveness, decreased. Similarly, García-Segovia et al. [6] also found that the hardness value decreased as the temperature and time increased due to starch gelatinisation during the cooking process.

The share and proportions of basic chemical ingredients decide not only on the nutritional value but, also, on the consumer attractiveness. Data on the chemical composition of medium-ground sausages are presented in Table 2. Analysing the basic chemical composition of medium-ground sausages produced, there was found a statistically significant (*p* < 0.05) impact of the smoking variant used on the water, protein and fat contents in this product but only for Country sausages. With the smoking variant B used in the Country sausage technological process, there were found statistically significant (*p* < 0.05) higher water and protein contents and a lower fat content compared to the amount of these ingredients determined in this product when using the smoking variant A. The contents of these ingredients in other sausages did not vary depending on the smoking variants used and were at similar levels for particular sausages.

The water content in meat products is mainly a result of its concentration in meat and fat raw materials. In the technological processes, water loss occurs. It results in the production yield being lower than 100% [36,37]. Products with excessive water contents are usually characterised by lower nutritional and sensory values and lower durability. Of the tested sausages, the lowest water content (53.12%) was determined in Country sausage and the smoking variant A used, while the highest water content (63.59%) was found in Bieszczady sausage and the smoking variant A used.

From a nutritional point of view, protein is the most important ingredient in meat. In meat and meat products, the content of high-quality protein is usually in the range of 10–20%. Lean meat can contain 20–24% of this ingredient [38,39]. The average protein content in the analysed sausages, considering the temperature of the furnace, upper and lower smoking chamber and the length of smoking time, ranged from 15.03–17.72%.

Fat is the main energy component of meat products. It also acts as a carrier for some hormones and vitamins (A, D, E and K). In addition, fat has a positive effect on the sensory properties of meat products—mainly, on smell and juiciness [40]. The lowest fat content (approx. 17%) was determined in Bieszczady sausage, while the highest amount of this ingredient was recorded in Country sausage with the smoking variant A used. Referring to the research carried out by Szmańko et al. [41] on the selected sausages, one can see a similar basic chemical composition to that determined in traditional medium-ground sausages in the author’s own research (Table 2). For example, the average protein content in Silesian, Krakow and frankfurter sausages determined by the cited authors was at the level of 11.29–19.51%, water at the level of 52.40–65.95% and fat at 15.61–3.70%. In studies conducted by Halagard et al. [42] on selected sausages from traditional and conventional productions, it was shown that the average water content ranged from 44.73 g/100 g to 60.14 g/100 g, proteins between 11.35 g/100 g and 24.3 g/100 g and fat from 21.80 g/100 g to 26.39 g/100 g. Grabowska et al. [43] in their research, on the other hand, obtained a higher average protein content in traditional smoked products (21.26%) and in local products (20.06%) compared to the amounts of these ingredients determined in sausages in the author’s research (Table 2). In turn, Halagarda et al. [44], analysing hams from traditional and conventional productions, showed that traditional hams are characterised by the highest nutritional value among the analysed product groups. The average water, protein and fat contents in traditional hams were, respectively: 63.4%, 25.5% and 8.56%), while in hams from conventional production, respectively: 73.1%, 16.6% and 5.81%. Research conducted by Lucarini et al. [45] on traditional Italian hams showed different chemical composition values. The cited authors determined the average protein content at 17%, water at 69.7% and fat at 9.6%.

It is well-known that some of the smoke components can influence meat product colours [46]. The results of the analysis regarding the impact of the smoking variant (differing in the temperature of the furnace, top and bottom of the smoking chamber and length) on the colour of the finished product are presented in Table 3. A statistically significant (*p* < 0.05) influence of the smoking variant on the brightness of the colour was found but only in the case of Bieszczady sausage. A lighter colour of Bieszczady sausage was obtained when the smoking variant B was used. The smoking variants used in the technological sausage process did not have a statistically significant effect on the differentiation of the colour parameters a* and b* of these products. Škaljac et al. [47], analysing the influence of smoking in traditional and industrial conditions on the colour of “Petrovská klobása”, showed that smoked sausage in traditional conditions has a statistically lower (*p* < 0.05) red (a*) proportion at the end of the drying-off period. In addition, the cited authors showed that the colour of smoked sausage under traditional conditions (L*, a* and b*) did not differ in a statistically significant way (*p* > 0.05) at the end of the storage period compared to smoked sausage under industrial conditions. Gomez and Lorenzo [48] and Ledesma et al. [46] found in their research that L*, a* and b* values of dry, fermented sausages decreased during the smoking, drying and ripening processes. In turn, Yang et al. [38] obtained lower (*p* < 0.05) L* values and higher a* and b* values for smoked sausages compared to roasted sausages. Sen et al. [49] found that an increase in the final core temperature of heating meat products intensifies the lightness L* on the cross-section and decreases the redness a*.

## 3. Materials and Methods

### 3.1. Preparation of Sausages

**Figure 1:**
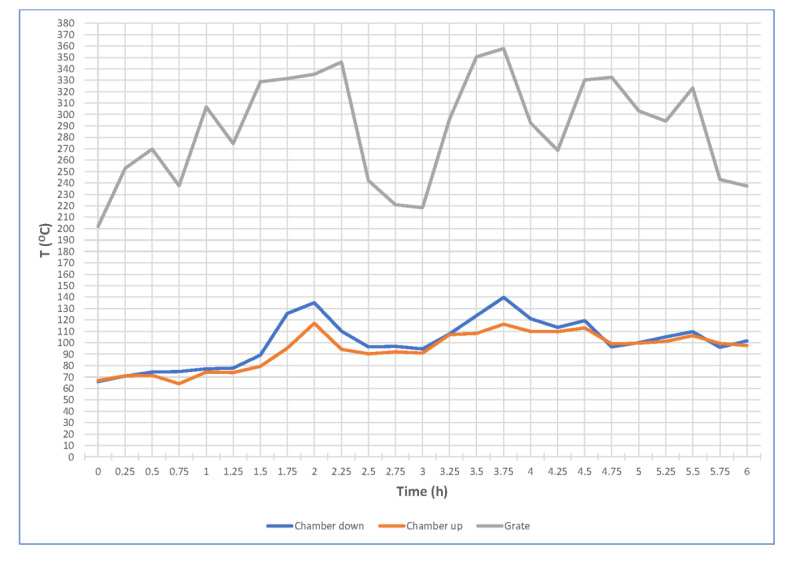
The temperature values (grate, top and bottom of the smoke chamber) obtained during smoking of medium ground sausages in variant A.

**Figure 2:**
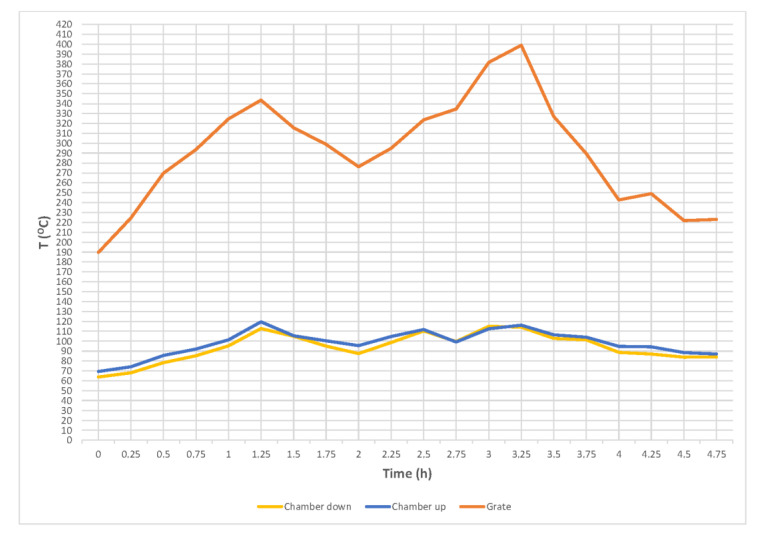
The temperature values (grate, top and bottom of the smoke chamber) obtained during smoking of medium ground sausages in variant B.

Meat and fat raw material for sausage production were obtained from the partition of pork and beef carcasses. The partition into pieces and the cutting out of fine meat for sausage production took place in refrigerated conditions (room temperature up to 12 °C). Dry curing of fine meat was used by mixing it with so-called pickling salt (99.4 kg of salt—NaCl and 0.6 kg—NaNO_2_) and leaving under refrigeration for up to 72 h. There was used about 2% addition of pickling salt proportionally to the meat. Then, the raw material was ground in a meat grinder (JR-120 Servotech, Shijiazhuang, China) using 10-mm screens. All ingredients ground in the meat grinder were mixed in a vacuum mixer (ZJB-340 Servotech, Zamosc, Poland) until they were evenly distributed. Spices were added while mixing. The natural casings (20–30 mm in diameter) were filled with a stuffer (Heinrich Frey Maschinenbau, Herbrechtingen, Germany) and left for about 2 h to dry the casing surface and deposit the ingredients in the bar. Smoking was carried out in a traditional smoking chamber with an indirect furnace using two variants (A and B) of this process, differing in temperature values, grate, top and bottom of the smoking chamber and the length of smoking time (Figure 1 and Figure 2). The critical temperature of the formation of polycyclic aromatic hydrocarbons (PAHs) (425 °C) was not exceeded in the furnace. Control tests on the PAH contents were carried out on sausage samples in an accredited laboratory. The results of these tests indicated that, with such a process of burning wood in the furnace (dry hardwood and wood chips used) and the correct construction of the chamber (the furnace with air supply), the level of PAHs not only did not exceed the value of the new legal regulations [50] but the results were much lower (by over 50%) than the requirements of the new regulations, despite the fact that the temperature in the chamber was relatively high, especially during baking (90−95 °C). Then, the sausages were cooled to a temperature below 8 °C inside the bar.

The tests were carried out on three types of medium-ground sausages:

1. Country sausage. Ingredients: pork (100 g of the product was made from 122 g of meat), salt, pepper and garlic.
2. Home sausage. Ingredients: pork (100 g of the product was made of 110 g of meat), salt, pepper and garlic.
3. Bieszczady sausage. Ingredients: pork and beef (100 g of the product was made from 98 g of pork and 27 g of beef), salt and natural spices.

### 3.2. Texture Measurement

Two samples from 10 batches of each type of sausage, produced using two smoking variants (A and B) were taken for testing (120 samples in total). Before the analyses were carried out, the material was stored in refrigerated conditions (temperature 0–4 °C). Subsequently, 20-mm cube samples were cut out, and the texture was determined using a Brookfield Texture-CT3-25 texture meter using a cylindrical probe (TA25/1000) of 50.8 mm in diameter and 20 mm in length. The TPA (texture profile analysis) test was used. Each sample was subjected to a double-compression test of up to 50% of their height. The texture meter was coupled with a computer with the Texture Pro CT program installed, through which the texture parameters were determined, i.e., hardness, cohesiveness, springiness, rigidity, resilience, chewiness, gumminess and adhesiveness. Hardness 1—the maximum force of the first compression, expressed in (N). Hardness 2—the maximum force of the second compression, expressed in (N). Rigidity 5—force required to deform a sample to a distance of 5 mm of the first compression. Rigidity 8—force required to deform a sample to a distance of 8 mm of the first compression. Springiness—it can be defined as the elasticity expressed in (mm); it is the rate of return of the tasted sample from the deformed state to the initial state. Cohesiveness—the area of work during the second compression divided by the area of work during the first compression. Adhesiveness—is the force required to separate the sample surface from the compressive plate surface in contact with it. Resilience—the ability of the product to return to its original from after the first compression. The ratio of the time needed to obtain the maximum deformation obtained during the second compression of the sample to the time necessary for maximum deformation during the first compression. Gumminess—force required to disintegrate a semisolid product to a state ready for swallowing. Chewiness—energy needed to chew a solid food until it is ready for swallowing, expressed in (mJ).

### 3.3. Chemical Composition

To determine the chemical composition of sausages (water, fat and protein content), the samples were ground in a laboratory meat grinder using a 4-mm mesh. The water content was determined in accordance with PN-ISO 1442:2000 [51]. The protein was determined using the Kjeldahl method, according to which, the determined nitrogen content was converted into protein, according to PN-75/A-04018 [52]. The fat content was determined using the Soxhlet method in accordance with PN-ISO 1444:2000 [53].

### 3.4. Colour Measurement

Instrumental colour measurement was performed on the cross-section of sausages in the CIE L*a*b* system using an electronic colourimeter NR20XE from EnviSense (light source D65, measuring head hole 20 mm and calibration with white standard: L*—99.18, a*—0.07 and b*—0.05). It used 45°/0° measuring geometry. During the measurement, the colourimeter was connected to a computer in which the CQCS3 ver. 3.4 EN software was installed. In this system, L* means brightness, which is a spatial vector, while a* and b* are trichromatic coordinates, where positive a* values correspond to red, negative—green, positive b*—yellow and negative b*—blue.

### 3.5. Statistical Analysis

All measurements were performed in triplicate. After being grouped, the obtained results were subjected to statistical calculations. Table 1, Table 2 and Table 3 contain average values ($\bar{x}$) and the standard error (SE) of particular features. To determine the significance of the impact of the smoking variant (A and B) on the differentiation of particular characteristics of the sausages analysed, one-way analysis of variance (ANOVA) was used. The significance of differences between means (*p* < 0.05) was determined by Tukey’s post-hoc test (RIR—reasonable significant difference). All statistical calculations were made in Statistica, version 13.

## 4. Conclusions

The study showed a statistically significant (*p* < 0.05) influence of the smoking variant on the differentiation of texture parameters such as cycle hardness 1 and 2, rigidity, gumminess and chewiness but only in the case of Bieszczady sausage. Higher values of these features were found when the smoking variant A was used for smoking in the technological process of Bieszczady sausage. When the smoking variant B was used in the technological processes of Home and Country sausages, slightly lower values of hardness, gumminess and chewiness were obtained; however, the differences for these features were not statistically significant. In addition, when using the smoking variant B in the technological process of Country sausage, statistically significant (*p* < 0.05) higher values of water and protein contents and a lower fat content were found compared to the amount of these ingredients determined in this product when using the smoking variant A. A statistically significant (*p* < 0.05) influence of the smoking variant on the colour brightness was also found but only in the case of Bieszczady sausage. A lighter colour of Bieszczady sausage was obtained when the smoking variant B was used. The smoking variants used in the technological processes of the sausages did not have a statistically significant influence on the differentiation of the colour parameters a* and b* of these products. The variant B was the more preferable variant of smoking to improve the texture parameters of all tested sausages and the chemical composition but only in the case of the Country sausage. However, the smoking parameters used in variant A influenced the darker colour of only the Bieszczady sausage.

## Figures and Tables

**Table 1:** Texture parameters of medium-ground sausages (2 samples × 10 batches × 2 variants × 3 types of sausages = 120 sausage samples) (average values ($\bar{x}$) ± SE).

Specification	Variant of Smoking	Sausage		
		Country	Home	Bieszczady
Hardness 1 (N)	A	19.48 ± 5.73	17.84 ± 4.49	27.10 ^A^ ± 7.68
	B	16.61 ± 2.75	14.95 ± 1.54	16.85 ^B^ ± 1.57
Hardness 2 (N)	A	17.73 ± 6.70	16.48 ± 7.07	24.66 ^A^ ± 6.64
	B	14.66 ± 2.65	13.78 ± 1.69	15.49 ^B^ ± 1.36
Rigidity 5 (N)	A	6.38 ± 0.80	5.95 ± 0.48	9.18 ^A^ ± 0.82
	B	6.75 ± 0.88	6.03 ± 0.60	6.65 ^B^ ± 0.55
Rigidity 8 (N)	A	11.75 ± 1.53	10.68 ± 0.84	16.99 ^A^ ± 1.32
	B	12.88 ± 2.33	11.00 ± 1.17	12.36 ^B^ ± 1.25
Springiness (mm)	A	6.79 ± 1.03	6.86 ± 1.47	7.25 ± 1.35
	B	6.03 ± 0.27	6.71 ± 0.39	6.71 ± 0.30
Cohesiveness	A	0.51 ± 0.05	0.51 ^A^ ± 0.02	0.54 ± 0.03
	B	0.48 ± 0.03	0.56 ^B^ ± 0.03	0.56 ± 0.02
Adhesiveness (mJ)	A	0.26 ± 0.05	0.18 ± 0.08	0.30 ± 0.04
	B	0.24 ± 0.06	1.65 ± 0.07	0.68 ± 0.09
Resilience	A	0.20 ± 0.03	0.22 ^A^ ± 0.02	0.23 ± 0.03
	B	0.20 ± 0.02	0.26 ^B^ ± 0.02	0.25 ± 0.02
Gumminess (N)	A	9.19 ± 3.12	9.00 ± 3.69	14.47 ^A^ ± 3.25
	B	8.06 ± 1.63	8.32 ± 1.09	9.39 ^B^ ± 0.99
Chewiness (mJ)	A	67.93 ± 15.60	66.43 ± 18.85	108.58 ^A^ ± 21.76
	B	48.84 ± 11.33	55.96 ± 9.39	63.10 ^B^ ± 8.14

^AB^—Different letters next to the mean in columns mean statistically significant differences at the level of *p* < 0.05.

**Table 2:** Basic chemical composition of medium-ground sausages (2 samples × 10 batches × 2 variants × 3 types of sausages = 120 sausage samples) ($\bar{x}$ ± SE).

Specification	Variant of Smoking	Sausage		
		Country	Home	Bieszczady
Water (%)	A	53.12 ^A^ ± 8.64	57.08 ± 4.71	63.59 ± 2.79
	B	62.52 ^B^ ± 1.40	55.13 ± 2.25	63.45 ± 0.64
Protein (%)	A	15.25 ^A^ ± 2.38	16.01 ± 1.97	17.72 ± 0.93
	B	17.19 ^B^ ± 0.39	15.03 ± 0.66	17.48 ± 0.18
Fat (%)	A	30.03 ^A^ ± 5.05	25.17 ± 6.27	17.47 ± 3.62
	B	18.32 ^B^ ± 1.73	27.85 ± 3.02	17.12 ± 0.78

^AB^—Different letters next to the mean in columns mean statistically significant differences at the level of *p* < 0.05.

**Table 3:** Colour parameters of medium-ground sausages (2 samples × 10 batches × 2 variants × 3 types of sausages = 120 sausage samples) ($\bar{x}$ ± SE).

Specification	Variant of Smoking	Sausage		
		Country	Home	Bieszczady
L (%)	A	63.78 ± 4.01	66.38 ± 1.92	56.57 ^A^ ± 2.10
	B	63.76 ± 3.22	67.10 ± 1.17	60.64 ^B^ ± 0.79
a	A	5.00 ± 1.94	6.75 ± 1.44	11.52 ± 1.30
	B	7.45 ± 0.80	6.94 ± 0.66	11.05 ± 0.78
b	A	10.85 ± 2.00	11.38 ± 1.26	10.79 ± 0.38
	B	11.59 ± 0.12	11.90 ± 0.66	11.07 ± 0.34

^AB^—Different letters next to the mean in columns mean statistically significant differences at the level of *p* < 0.05.
